# Dream-chasing mindscapes: an inquiry into the psychological change process of postgraduate entrance examination candidates

**DOI:** 10.3389/fpsyg.2026.1801825

**Published:** 2026-05-20

**Authors:** Chen Xiaoman, Lu Zihui, Shao Juan

**Affiliations:** 1 School of Marxism, Xihua University, Chengdu, Sichuan, China; 2 School of Educational Sciences, Sichuan Normal University, Chengdu, Sichuan, China; 3 The Open University of Sichuan, Chengdu, China

**Keywords:** candidates, emotional regulation, postgraduate entrance examination, psychological change, social support

## Abstract

As a pivotal pathway for talent advancement in higher education, the postgraduate entrance examination not only shapes the depth of individuals' academic engagement and the breadth of career development, but also carries candidates' distinctive trajectories of psychological growth across the long preparation cycle. To explore these trajectories during exam preparation, this study focuses on postgraduate entrance examination candidates and adopts a qualitative research design. Analysis of candidates' preparation experiences shows that their psychological States change dynamically over time: in the early stage, they are often marked by confusion. In the middle stage they tend to slip into anxiety, and in the late stage some candidates respond with composure whereas others become so tense under pressure that they approach psychological breakdown. When confronting confusion, anxiety, and a range of other difficulties, candidates regulate themselves through goal orientation, emotional regulation, and social support, thereby preparing for the examination in a better condition. The preparation experience and psychological growth accumulated by these candidates provide valuable points of reference for subsequent cohorts. Accordingly, drawing on the interview findings, this paper proposes stage-specific recommendations in the hope of offering practical and effective guidance and support for more candidates.

## 1 Introduction

With the continuous development of higher education in China, university enrollment has expanded, the stock of college students has increased, and the number of graduates has risen year by year. According to projections jointly released by the Ministry of Education and the Ministry of Human Resources and Social Security, the number of university graduates in 2025 is expected to reach a record high of 12.22 million (Ministry of Education of the People's Republic of China, 2024). As large cohorts of graduates enter the labor market, the competitive advantage of a bachelor's degree has gradually weakened. To enhance their competitiveness in the labor market, increasing numbers of undergraduates choose to take the postgraduate entrance examination after graduation. According to statistics from the Ministry of Education, the number of applicants increased from 1.77 million in 2016 to 3.88 million in 2025 (Ministry of Education of the People's Republic of China, 2015; EOL China, 2026), while the number of master's admissions in 2025 (excluding recommended admissions and separate examinations) was 872,200 (EOL China, 2024). This means that 77.52% of candidates faced non-admission. Such a high non-admission rate places candidates under tremendous psychological pressure during preparation. Intense pressure often gives rise to negative emotions such as tension, anxiety, and depression, affects sleep and healthy physical and mental development, and interferes with candidates' normal performance in the examination.

Existing studies on the postgraduate entrance examination mainly concentrate on the following strands. With respect to preparation experiences, Xiong Yinwan analyzed the preparation experiences of candidates applying for education majors from four dimensions—preparation choice, preparation learning, preparation capital, and preparation experience—and argued that the decision to prepare is jointly driven by internal and external forces, with preparation choices shaped by both rational and non-rational factors (Xiong, 2021). Zhang Libing contended that the successful preparation experiences of undergraduate nursing students could be summarized into six themes—fear of the examination, perseverance, considerations in selecting institutions, preparation planning, coordination and adaptation, and stress relief—and pointed out that successful candidates usually show relatively firm perseverance and more reasonable review plans, although they still undergo various forms of pressure (Zhang and Lu, 2023). Shen Lujun reported that the vast majority of students who had taken teacher-training postgraduate entrance examinations experienced examination anxiety; in the pre-examination stage, 82.7% reported anxiety, of whom 24.2% reported high levels of anxiety (Shen, 2006). Chen Luying argued that intensive studying during preparation is often accompanied by loneliness: such loneliness stems not only from the solitary, self-reliant experience of preparing in relative social isolation, but also from the difficulty of finding strong-tie support and resonance in everyday life, which in turn produces a sense of fragility and isolation (Chen and Lin, 2024). Guan Huiyu likewise observed that candidates often feel lonely because of social isolation and insufficient external support, yet may also derive fulfillment and self-affirmation from focused preparation, thereby forming an experience in which positive and negative emotions are intertwined (Guan and Cheng, 2024). Because the postgraduate entrance examination often coincides with internship periods, Candidates are subjected to the dual pressures of the examination and the internship; academic pressure and internship fatigue, self-doubt and fear of failure, employment pressure, and a lack of information and resources all bring varying degrees of negative experiences and emotional fluctuations (Wang et al., 2024).

With respect to motivations for taking the postgraduate entrance examination, the decision reflects the joint influence of multiple factors. Tian Haoran proposed that students may choose to prepare either out of rational considerations, out of emotional attachment, or passively under external influence (Tian and Gong, 2023). Liu Tianjun found that a major motivation for taking the examination is to avoid temporary employment pressure, and that nine factors—gender, academic performance, participation in research, parental attitudes, family income, institutional type, major satisfaction, the number of classmates preparing for the examination, and perceived employment pressure—significantly affect students' willingness to take the examination (Liu, 2013). Ji Xiangpei argued that university graduates regard the postgraduate entrance examination as a pathway to higher social status, hoping to enhance their cultural capital—especially institutionalized cultural capital in the form of educational credentials—through higher-level schooling and thereby obtain occupations with greater economic returns (Ji and Li, 2021). Zhou Tiantian noted that some college students gradually realize after entering university that the atmosphere and prospects of top-tier universities differ substantially from those of ordinary undergraduate institutions, and therefore aspire to pursue graduate study at better universities as a means of proving themselves (Zhou and Miao, 2023). Jung further proposed that students' decisions to pursue a master's degree are mainly influenced by factors such as gender, age, and family socioeconomic status (Jung and Lee, 2019).

Research on the master's postgraduate entrance examination has largely focused on preparation experiences and motivations, while paying relatively little attention to the psychological states of candidates during preparation. As the central actors in this examination, candidates and their preparation experiences, processes, pressures, and psychological states have not received sufficient attention. The lack of inquiry into this group's preparation experiences and patterns of psychological growth makes it difficult for existing research to provide effective guidance or to meet candidates' practical needs during preparation. Accordingly, this study interviewed 31 students who had previously prepared for the postgraduate entrance examination, explored the trajectories of their psychological changes across different preparation stages, and examined the specific measures they adopted to cope with pressure and difficulty, with the aim of providing psychological support for future candidates.

## 2 Theoretical foundation

Psychological resilience, also referred to as resilience or mental toughness, emphasizes an individual's ability to achieve efficient adaptation and recovery when facing challenges. This kind of capacity is a dynamic and mutable trait manifested by individuals in stressful situations; namely, it constitutes a process of dynamic adaptation and growth when they cope with adversity (Wang, 2018). It mainly includes three elements: emotion regulation, goal orientation, and social support. During the postgraduate entrance examination process, candidates face challenges such as memory pressure, goal confusion, and progress anxiety. The elements of psychological resilience, including emotion regulation, goal orientation, and social support, provide corresponding solutions to these problems. For instance, goal orientation can help quickly sort out information about universities and select those that suit the individual. Emotion regulation can help relieve anxiety. Social support can offer information sharing, emotional comfort, and resource mutual assistance to candidates, building a study community and a multi-dimensional support network, and assisting candidates in continuously obtaining motivation and direction throughout the postgraduate entrance examination journey. Coping effectiveness in turn influences levels of psychological resilience: effective coping strengthens resilience and makes candidates more capable of withstanding pressure, whereas inadequate coping weakens resilience and makes them more likely to fear difficulties and give up.

In the foundational stage of preparation, goal ambiguity and information anxiety are salient, and goal orientation and emotional regulation are therefore key to activating resilience. At this stage, goal orientation reduces confusion and transforms anxiety by clarifying preparation direction, while emotional regulation helps prevent negative emotions from obstructing action; together, they lay the foundation for resilience. In the strengthening stage, progress pressure and self-doubt intensify, and emotional regulation and social support become important supports for further strengthening resilience. Emotional regulation can guide candidates to transform self-denial into constructive self-improvement, while social support reduces self-doubt through access to resources and emotional resonance. Social support serves as a protective factor for psychological resilience, and sound social support can create favorable conditions for individuals to actively cope with various challenges (Sang et al., 2016). In the final stages of exam preparation, candidates face dual challenges of recurring anxiety and fluctuating efficiency, pushing psychological stress to its peak. Through the combined effects of social support, emotional regulation, and goal-oriented strategies, students can develop the mental resilience needed to approach exams with composure. From the early stage of preparation to the eve of the examination, the cultivation of psychological resilience is not a linear process; rather, it relies on stage-specific adaptive strategies through the coordinated functioning of goals, emotions, and social support to achieve further strengthening.

## 3 Research design

### 3.1 Research method

Psychological changes during preparation for the postgraduate entrance examination are influenced by multiple factors, including knowledge demands, examination policies, family, school, environment, and individual differences, and therefore display stage-specificity and heterogeneity. Research on preparation experiences and psychological growth among postgraduate entrance examination candidates has thus often adopted qualitative methods both in China and internationally. In the implementation of such studies, and in order to strictly comply with research ethics and safeguard the lawful rights and interests of interviewees, written consent forms are generally signed by participants. On this basis, the present study likewise adopts a qualitative research paradigm and takes thematic analysis as its core qualitative method. The study strictly follows a stepwise coding procedure consisting of three stages:

The first step is open coding. Open coding refers to the process of meticulously examining and dissecting the research textual materials word by word and sentence by sentence, thereby forming a list of highly divergent themes. Eventually, the core themes hidden in the deep layers of the materials are extracted and presented through this coding method (Feng, 2018), which involves breaking down the collected interview data word by word and sentence by sentence, discarding invalid information, extracting initial concepts related to the experience of preparing for the postgraduate entrance examination and psychological growth, and categorizing similar initial concepts to form preliminary categories, ensuring the objectivity and comprehensiveness of the coding. All coding work was conducted manually, and no software was used to assist in the coding process. The research first sorted through the text materials of the 31 interviewees word by word and sentence by sentence to fully grasp the core content of the interviews. On this basis, it discarded invalid information unrelated to the experience of preparing for the postgraduate entrance examination and psychological growth, and screened out valuable relevant content. Then, it broke down and coded the screened content, extracted the corresponding initial concepts, and categorized and organized similar initial concepts to form preliminary categories. Finally, through sorting and summarizing, it concluded three core themes and six sub-themes related to “initial confusion, mid-term anxiety, late-term collapse and composure” (Table 1).

**Table 1:** Coding process example.

Raw data excerpt (interview quote)	Initial code	Sub-theme	Overarching theme	Frequency of occurrence
“I'm torn between choosing a double first-class university or a regular undergraduate program, and I'm not sure which institutions are more welcoming to cross-disciplinary applicants.” (F3B3ZB)	Confusion over institutional tiers, ambiguous cross-institutional exam compatibility; difficulty level of target institutions' entrance exams; unclear self-assessment of learning capacity	Confusion in target school selection	Initial confusion	Twenty-six
“After three months of study, I've reviewed all the material thoroughly. But when I started the second round, I found the earlier content was completely unfamiliar, and I couldn't apply it flexibly to solve problems.” (M1A1ZB)	Excessive knowledge retention pressure leads to output difficulties; internships encroach on study time; doubts arise regarding admission to the desired target institution	Anxiety over knowledge mastery; anxiety caused by interfering factors; lack of confidence in exam preparation	Intermediate anxiety	Twenty-three
“As the exam approaches, my study efficiency keeps dropping, and the more I study, the more self-doubt and anxiety I feel.” (F1C1ZB)	Self-doubt, low learning efficiency, and poor exam preparation	Low learning efficiency as the final examination approaches	Later crash	Seventeen
“The notion of stress-free post-study is an illusion, but what predominates is the anticipation of seeing the fruits of one's labor through this exam.” (F3D3ZB)	Through persistent self-adjustment and early-stage dedication, one can then simply follow the exam schedule with patience	In the later stages, I approached the exams with greater composure	Be calm in the later stage	Fourteen

Second, axial coding was conducted on the basis of the preliminary categories generated through open coding to excavate the internal relationships among them, sort out causal and subordinate relations, integrate dispersed categories into several core categories, and build a category system. Third, selective coding was employed to extract from the core categories the principal category capable of integrating the entire study, clarify the logical relations among the principal category, the core categories, and the initial categories, and ultimately form central themes that fit the research topic. Theme generation was centered on the coding results and repeatedly checked and revised in light of the original research purpose and the core needs of research on preparation psychology. Content less closely related to the research topic was excluded, while common viewpoints and materials were integrated, resulting in three major themes and six subthemes that accurately reflect the core characteristics of candidates' preparation experiences and psychological growth. Throughout the research process, inductive analysis was used to analyze and excavate the collected materials, thereby constructing a dynamic analytical framework for the psychology of postgraduate entrance examination preparation.

This method repeatedly investigates and interviews the preparation phenomena and candidate behaviors under study according to research needs, and uses inductive analysis to analyze and excavate the collected materials so as to construct the model required by the research. In relation to candidates' preparation behaviors, this study selected candidates from different majors and backgrounds for qualitative interviews, analyzed their experiences during preparation, elucidated the causes and manifestations of psychological growth, and proposed optimization suggestions in the hope of improving the efficiency and quality of preparation and promoting candidates' psychological well-being.

### 3.2 Data sources

With the goal of precisely capturing psychological changes across the entire preparation process, this study used a combination of purposive sampling and snowball sampling to select interviewees. In the purposive sampling stage, the study focused on students who had participated in the postgraduate entrance examination and screened eight initial interviewees. Thirteen potential initial interviewees were preliminarily identified, of whom one was excluded because the preparation period was shorter than 3 months and another because changes in psychological state were not salient. Ultimately, 11 preliminary interviewees were confirmed. These 11 participants covered subgroups across the humanities, sciences, and engineering, Double First-Class universities, ordinary undergraduate institutions, cross-major applicants, and non-cross-major applicants, thus laying a targeted foundation. Due to the limited scope of the researchers' contact, further expansion of the sample was achieved through snowball sampling, requiring the initial respondents to recommend similar individuals and supplement the uncovered differences in dimensions. At the same time, three consecutive rounds of new interviews were set up, with 5–7 people in each round. If no new types of test-taking psychological changes emerged, it would be considered a termination criterion for theoretical saturation. After completing the fifth round of interviews, which included the initial 11 participants, no new psychological change characteristics, emotional expression dimensions, or psychological adjustment methods were found in the subsequent three rounds of interviews. Thus, it was officially determined that theoretical saturation had been reached and sampling was stopped. A total of 42 people were included, among whom four had a better later state and no significant changes in mindset during the preparation process, two had less than 3 months of preparation and did not have a phased nature, two dropped out of the exam, and three had worse resilience after taking the exam. These 11 were not included in the sample size. Finally, the sample size was determined to be 31 people.

### 3.3 Organization of interview materials

This paper adopts a five-level coding scheme for interviewees. The first level denotes gender: *M* for male and *F* for female, directly distinguishing gender. The second level denotes the target institution: 1 = top-tier universities (985/211), 2 = Double First-Class discipline universities, 3 = non-Double First-Class universities, and 4 = in-service candidates, reflecting differences in candidate sources. The third level denotes major: A = Marxist Theory, B = Sociology, C = Education, D = Psychology, and E = Management, covering five majors so as to avoid homogeneity. The fourth coding layer represents the number of postgraduate entrance examination attempts, where “1Z” denotes a first attempt, “2Z” denotes a second attempt, and “3Z” denotes three or more attempts, thus clarifying the specific number of exam preparations. The fifth level denotes whether the candidate switched majors: B = same-major application and K = cross-major application, distinguishing application types. See Table 2 for details.

**Table 2:** Coding Information of interviewees.

Group	Code	Code	Code	Code	Code
1	M1A1ZB	F1B2ZB	F3B3ZB	M3A1ZB	F3B3ZB
2	M1B1ZK	F1C3ZK	F2A2ZK	M3B1ZK	F3C2ZK
3	F1C1ZB	M2A1ZB	F2B2ZB	F3C1ZB	M3D2ZB
4	F1D1ZK	F3B2ZK	F2C3ZK	F3D1ZK	M3E2ZK
5	F1E2ZB	F2C1ZB	M2D3ZB	F3E2ZB	F3B1ZK
6	F1A2ZK	F2D1ZK	F3B1ZK	F3A2ZK	F3A1ZB
7	F3D3ZB				

## 4 A qualitative study of the psychological growth process of postgraduate entrance examination candidates

The postgraduate entrance exam preparation for candidates is a protracted process, which mainly consists of three phases: the initial stage, the intermediate stage, and the final stage. Across these stages, candidates' psychological states change dynamically—from confusion and apprehension in the early stage, to anxiety and helplessness in the middle stage, and finally to a condition that hovers near breakdown in the late stage before gradually learning to face the process with calm.

### 4.1 Psychological changes during preparation

1. Early-Stage Confusion

In the early stage of preparation, , the selection of a target institution is one of the major problems candidates face. Determining a target institution is the result of the interweaving and joint effects of multiple factors. Candidates need to objectively assess their own learning ability and knowledge base while also considering such factors as future career planning, institutional tier, examination difficulty, applicant-to-admission ratio, the broader social environment, and family economic conditions. Accordingly, the selection of a target institution, together with the matching selection of a major, involves numerous complex factors and bears significantly on candidates' future development; it therefore becomes a major source of difficulty. One interviewee noted that,

“due to family finances, the costs of graduate study were an important factor in choosing a university, while the location of the university and the ranking of the major also had to be considered, making it difficult to satisfy all conditions simultaneously” (M1A1ZB).

One interviewee noted that,

“The initial confusion stems from an overly broad selection range of target universities, requiring comprehensive comparisons and gradual screening. The sheer number of options paradoxically intensifies indecision and uncertainty” (M3A1ZB, F1D1ZK).

2. Mid-Stage Anxiety

The postgraduate entrance examination is a protracted battle. After determining the target university and gathering all the necessary reference materials, students enter the mid-term stage of the exam, which is characterized by a long time span and heavy tasks. During this period, postgraduate entrance examination candidates are confronted with challenges from all aspects.

(1) Pressure from Knowledge Memorization

The knowledge system of humanities and social sciences is complex and extensive, and candidates often face severe challenges during the preparation for the postgraduate entrance examination. Firstly, the volume of knowledge to be memorized is huge. The postgraduate entrance examination preparation tasks are arduous, usually involving postgraduate political science, postgraduate English, and two professional courses. Although some special majors only require one course, the examination content is still quite extensive. Whether focusing on a single subject or handling two subjects simultaneously, students are under considerable pressure. Secondly, knowledge is prone to confusion or forgetting. Most candidates have less than a year for preparation, while a few have more than a year. The long preparation period requires candidates to repeatedly and persistently memorize the knowledge. Coupled with the vast amount of knowledge, the knowledge points are easily confused or forgotten, necessitating continuous consolidation and review to maintain the intensity and accuracy of memory. This poses a significant test to the candidates' memory and endurance. Third, knowledge output is difficult. Examinations in the humanities and social sciences are largely composed of subjective questions, requiring candidates to reproduce memorized knowledge accurately and completely on the answer sheet. Candidates must both remember knowledge points and apply them flexibly, which raises the demands placed on the proficiency and accuracy of memorization. One interviewee remarked that by the middle stage,

“I would forget what I had learned earlier once I started learning new material; I didn't even know what I had studied, and I began to doubt my learning ability” (F2D1ZK).

“Another said that during the summer vacation they had basically gone through the theoretical knowledge once and begun trying practice questions, only to find that they could not answer them at all and felt as though all of their previous study had been in vain” (F1B2ZB).

A cross-major humanities candidate likewise observed:

“Before I started preparing, I thought humanities was just a matter of memorizing things—it wasn't like the sciences, where if you don't know something, you simply don't know it. But once I began trying to produce answers, I realized that there was truly too much to memorize in the humanities. The content was enormous, both the study cycle and the review cycle were long, and forgetting what you learned earlier while moving on to later material was commonplace” (F1A2ZK).

(2) Excessive External Distractions

Preparation outcomes result from the joint effects of internal and external factors. While personal factors are certainly important, external factors are also non-negligible. Unavoidable factors such as changes in admissions policies, replacement of reference books, reduced quotas, employment, and family economic conditions can affect candidates' mindsets, obstruct or weaken their determination, and influence overall preparation status. Moreover, internships compress review time and fragment preparation time, which is unfavorable for concentrated study. The combined effects of these factors make the road to the postgraduate entrance examination even more arduous and impose greater psychological pressure on candidates. As one interviewee noted,

“once they arrived at their internship school in September, they had to learn classroom teaching and class management from their mentor teacher while also keeping up with specialized courses; time became fragmented and it was difficult to concentrate on studying” (M3D2ZB).

Another interviewee recalled that

“When the target institution released its admissions brochure and they saw that the reference books had changed, the first thought that flashed through their minds was that all of their previous studies had been wasted, and they immediately wanted to give up, feeling utterly desperate and shattered” (F1C1ZB).

Still another interviewee explained that

“When the time came in September to pay tuition after the new semester began, my parents did not have enough money, So I had to use money earned from earlier part-time jobs to pay tuition; yet I still needed to support themselves, and therefore had no choice but to work part-time while preparing for the examination” (F3B1ZK).

(3) Shaken Commitment

By the middle stage, candidates face more numerous and more severe obstacles. Under the influence of heavy learning tasks, complex and difficult-to-memorize knowledge points, monotonous study routines, time taken up by internships, and an uncertain employment environment, candidates' faith in persisting with preparation gradually begins to disintegrate. As a result, some candidates' willpower wavers, and their commitment to preparation is no longer as firm as it was in the early stage; some have their study plans disrupted, which affects preparation effectiveness and may even influence final outcomes. Accordingly, at this crucial stage, firm determination is of great importance: it enables candidates to resist temptation, remain steadfast in adversity, and proceed steadily according to plan. Only with strong resolve can candidates overcome obstacles and realize the dream of being “admitted.” One interviewee reflected that

“Some peers had already found jobs and others had given up on preparation; being the only one still persisting made them want to do the same as them—just find a job and be relieved” (F3D1ZK).

“I spent the entire summer vacation preparing for the postgraduate entrance exam on campus. During the daytime, I struggled with extremely poor learning efficiency, and at night, I was too anxious to fall asleep. Trapped in this vicious cycle, I seriously felt like giving up” (M3B1ZK).

“I was taking the postgraduate entrance exam for the second time. When I found out that my target university had cut the number of open admission spots due to more places being given to recommended students, and that one key specialized course now required four reference books instead of two, my hopes of getting accepted faded even more. Being a re-sit candidate, I had little room for mistakes, so I couldn't stop thinking about failing the exam. I started regretting choosing to try again, and it felt so difficult to keep persevering” (F2B2ZB).

3. Late-Stage Composure and Breakdown

In the final phase of postgraduate entrance exam preparation, candidates' psychological states are primarily characterized by two dimensions: anxiety and composure.

(1) Composure and Calmness

Having endured the protracted grind of postgraduate entrance exam preparation, candidates have not only undergone repeated mental adjustments internally but also withstood the trials of external distractions. They no longer rush to pursue high scores in mock examinations, but instead use mock tests to analyze how to allocate time when answering questions. They do not seek excessively high efficiency; rather, they focus on whether the small tasks of each day have been completed, advance their established plans step by step, and calmly wait for the examination to arrive. This state may not be one of total self-confidence, but it does enable candidates to face the final examination with composure, greater confidence, and greater courage. One interviewee noted that,

“By the end, they were no longer trapped in anxiety over whether they could be admitted; instead, they simply completed what needed to be done each day with peace of mind and looked forward to the examination arriving soon ”(M1B1ZK).

Another interviewee said that,

“In the last month before the examination, there was less anxiety than exhaustion; perhaps because they were simply too tired, they became somewhat numb, did not think too much, and only wanted the examination to end quickly so that they could finally rest” (F3A2ZK).

The interviews also suggest that candidates who had prepared seriously and steadily in the early stage—although they too experienced doubt, anxiety, and pressure—tended in the late stage to look forward even more eagerly to the examination, because they were eager to use it to prove a year of effort and commitment. By contrast, candidates who had not invested sufficient time earlier tended to become more anxious in the late stage and to regret not having started earlier or not having done what they should have done.

(2) Reduced Learning Efficiency

Physical health is the foundation of success and a prerequisite for sustained preparation. In the late stage, candidates have often experienced more than half a year of continuous high-intensity study. Especially near the end, all subjects require review time; time allocation is tight, mock exams are frequent, and candidates may study late into the night, resulting in extreme physical and mental fatigue. Long-term intensive study can lead to high energy depletion, insufficient sleep, and lack of exercise, making it difficult to concentrate. In addition, as the exam approaches, expectations and worries about outcomes peak; negative emotions such as anxiety and fear keep the mind in a high-stress state, lowering learning efficiency and impeding preparation. One interviewee described that,

“In the late stage, knowledge that was once familiar suddenly became unfamiliar; they had no learning state at all, did not want to study, and could not absorb material even when trying” (F3A1ZB).

Another said that,

“For reasons they could not explain, the more they memorized in the late stage, the less they could retain; their memorization capacity was nowhere near what it had been before, and because the amount remembered became larger, it also became more chaotic, forcing them to slow their pace” (M2D3ZB).

(3) Self-Doubt

Facing the impending examination, candidates may experience unprecedented tension and anxiety. As the examination nears, symptoms such as sleep deprivation and insomnia become more pronounced; learning efficiency is far lower than in the early and middle stages; memory weakens; retrieval of knowledge becomes difficult; and mock examinations follow one after another, placing the psychological state under severe strain. Candidates begin to doubt whether they are adequately prepared and worry that they will be unable to perform normally in the examination. The unknown and uncertainty intensify anxiety, interfere with learning progress, and weaken candidates' motivation and confidence. One interviewee said that

“The late stage was simply too overwhelming: they could not study, but also did not dare to relax, and in their minds, two “selves” argued every day—one telling them to give up and the other telling them to persist” (F2C3ZK).

Another candidate explained that,

“When confronted with several thick sets of specialized-course knowledge points, they broke down every day, crying while memorizing and memorizing while crying; their psychological state was extremely poor, and they even felt that perhaps they were not suited to the path of postgraduate entrance examination preparation at all” (M3E2ZK).

### 4.2 Analysis of the causes of psychological changes

During the initial stage of choosing schools, the middle stage of intense preparation, and the final stage of sprinting, the psychological states of postgraduate entrance examination candidates undergo significant changes, including confusion, anxiety, collapse, and composure. The underlying causes mainly focus on three core factors: goal orientation, emotion regulation, and social support. To clearly dissect how these factors influence different stages of the preparation process, the following text will conduct in-depth analysis by stage.

1. Root Causes of Candidates' Confusion in Selecting Target Postgraduate Institutions

At the beginning of the postgraduate entrance examination, the selection of the target university is the main cause of the initial confusion among postgraduate candidates: Firstly, the complexity of the target university selection. The selection of the target university not only needs to consider the internal factors, such as one's major, learning ability, career planning, etc., but also needs to consider various external factors such as the geographical location of the university, level, admission-to-application ratio, examination difficulty, reference textbooks, and family economic conditions for weighing the pros and cons, and making a choice that suits one's own development. The combination of many factors has greatly increased the difficulty of choosing a school, and postgraduate candidates are in a state of confusion and indecision. Secondly, the ambiguity and variability of the goals have also exacerbated the confusion among the candidates. Many students preparing for the postgraduate entrance examination did not have clear and firm goal orientations at the beginning of their preparation. They lacked in-depth thinking about what their core demands for pursuing a postgraduate degree were, and which field they wanted to deeply develop and grow in. They were unclear whether their purpose of pursuing a postgraduate degree was to enhance professional capabilities, pursue academic advancement, or to obtain a higher degree to enhance their employment competitiveness. This ambiguous goal orientation would leave postgraduate candidates without clear screening criteria when choosing universities, and they would be at a loss as to how to make a choice among numerous university information. The goal orientation of some postgraduate students is prone to change. When they learn that the questions in other schools or majors are simpler, and the professional ranking is higher, making it more conducive to employment, they lose their original intention to apply to the target university. The instability in choosing a goal leads them to constantly adjust their choices during the school selection process, making it difficult for them to form a fixed range of options. As a result, they become more and more confused in the repeated deliberations.

2. Analysis of the Causes of Mid-Stage Anxiety

Firstly, improper emotion regulation intensifies psychological internal strain. The reason why external interference factors are a major obstacle for postgraduate students during their mid-term preparation, and why knowledge memorization is voluminous, confusing, and difficult to output, lies in the fact that the candidates fail to adjust their mindset through effective emotion regulation. Instead, they get trapped in a cycle of amplifying negative emotions. Through interviews, it is known that most liberal arts candidates face difficulties such as high pressure in knowledge memorization, difficulty in output, and lack of flexibility in using knowledge to solve practical problems. This is not an individual issue but a widespread problem. However, some candidates directly elevate these problems that most people have to personal issues or even issues of their own insufficient ability. Candidates lack the ability to regulate their emotions in a positive and constructive direction, leading them to get trapped in a cycle of anxiety, internal strain, and low learning efficiency. The reasons why external factors such as target universities changing reference books, internships, and admission policies become problems for candidates' anxiety lie in the fact that these problems are beyond the candidates' ability to avoid and cannot be changed by themselves. Some candidates lack positive and proactive emotional regulation. Once a reference book is changed, they immediately fall into despair, feeling that everything they learned earlier has been in vain. As a result, they fail to use rational analysis—for example, recognizing that where the subject remains the same there must necessarily be connections between the new and old reference books—to reduce fear of the unknown. Nor do they promptly sort out the differences and links between old and new knowledge points so as to translate anxiety into concrete action. Instead, they allow negative emotions to spread and intensify, falling into fears of not having enough time, not being able to finish studying, and not being admitted. Some candidates have relatively few formal classes during preparation and are accustomed to studying in large uninterrupted blocks of time. When internships begin and time becomes fragmented, they become trapped in the fixed idea that they are unsuited to fragmented learning, rather than adjusting their way of thinking by making use of fragmented time or coordinating flexible time with internship supervisors. Some students have fewer courses during their preparation period and mainly spend their study time in a continuous manner. When the internship arrives, the time is divided, causing them to get stuck in their inherent mindset that is not suitable for fragmented learning and unable to let go. Instead of changing their mindset and using fragmented time for learning, they fail to coordinate with the internship teacher about flexible time, making it difficult to regulate their anxiety and leading to a loss of balance in their mindset and a decline in learning efficiency.

Secondly, insufficient social support undermines preparation efforts. Social support is a crucial pillar for postgraduate entrance exam candidates to sustain their learning motivation and maintain a steady study rhythm. It includes material support and emotional recognition from the family, as well as environmental guarantees and reasonable arrangements provided by schools. Adequate social support can alleviate candidates' anxieties and allow them to concentrate on their studies, acting as an indispensable psychological buffer and practical support system throughout the preparation period. However, in reality, some candidates receive significantly inadequate social support during their exam preparation:

At the family level, some candidates are constrained by their family's financial situation and lack the necessary material support. As a result, students have to work part-time to cover their expenses, which squeezes out the already tight study time. Some parents lack understanding of the postgraduate entrance examination and neither provide the necessary funds for living and studying nor offer emotional encouragement and recognition. They even often question the candidates' choices with statements like “The postgraduate entrance examination is useless” or “It's better to find a job earlier”.

“My family didn't support me at all to take the postgraduate entrance examination and hoped that I would start working earlier. However, even though I insisted on taking the exam, my father, who knew that I was taking the exam, still asked me every day if I had gone to do part-time jobs to earn money. It feels so exhausting” (F1C3ZK).

At the school level, the internship schedules of some institutions conflict with crucial preparation periods such as the later stages of the entrance examination, and there is no flexibility for adjustment, which disrupts the students' established review plans.

“Everyone else starts their internships in September, but our school begins with internships during the summer vacation and continues with internships even after the start of the new semester. This means that throughout the crucial review period, we are all engaged in internships, which really affects the review state” (F1E2ZB).

Additionally, the preparation hardware environment of some schools is weak. They not only lack dedicated study rooms for the entrance examination, but also have long-standing shortages of library seats, forcing students to spend extra effort competing for or finding study places, further increasing the burden of preparation. Another interviewee said that

“Throughout the summer vacation they kept looking for classrooms in which to study; the library was closed, and seats in the postgraduate-exam study room had to be contested every day, so that if one was slightly unlucky, the only remaining option was to return to the dormitory to study” (F2C1ZB).

The absence of social support not only puts candidates in a passive position with respect to time allocation, but also leaves them carrying a sense of loneliness produced by having no support at all. When they encounter difficult knowledge points or fall behind schedule, they may develop self-doubt, which gradually weakens preparation motivation and may even give rise to thoughts of giving up, thereby adversely affecting both the preparation process and the final outcome.

3. Reasons for Composure Vs. Breakdown in the Late Stage

In the late stage, candidates mainly display two distinct states. The first is composure and calmness. This state results from the joint effects of firm belief and timely intervention in, and adjustment of, negative emotions, which together lay the foundation for meeting the examination in a calm and collected manner. From the perspective of the practical path of goal beliefs, the strong identification and aspiration toward the target institution prompt candidates to form clear daily study goals and task lists. On the one hand, the clear task orientation avoids the blindness of the study process; on the other hand, by completing the daily tasks with high quality, candidates gradually accumulate their study achievements, and their mastery of knowledge gradually transforms from quantitative change to qualitative change. The effect of knowledge transformation is directly reflected in the mock tests, providing positive feedback that enables them to more clearly perceive the effectiveness of their study, thereby enhancing their confidence in facing the exam and providing psychological assurance for them to calmly meet the exam. Emotional regulation is the key to maintaining the candidate's study rhythm and avoiding a collapse in mentality, directly affecting whether the long-term accumulated knowledge can be transformed into the ability to perform on the exam. During the process of emotional regulation, candidates will use positive psychological suggestions and self-motivation to soothe their emotions, thereby dispelling negative emotions and concentrating their action drive. For example, one of the interviewees said:

“When feeling tired, tell yourself to hold on for a while until you reach the end. Use the experience of having overcome so many complex problems and mastered so many obscure knowledge points to strengthen confidence. When feeling anxious, think that many people have given up, and that I have persisted until now, which means I have won most of the battle” (F3B2ZK).

Ultimately, effective emotional regulation enables students in the later stages of the exam to still feel nervous even in the face of the upcoming final test, but they can always maintain an expectant, calm and stable mindset due to a clear understanding and firm recognition of their long-term efforts, which helps them remain confident and composed in the exam.

The second state is tension and breakdown. For some candidates, anxiety mainly arises from weakened goal orientation and emotional collapse caused by maladaptive emotional regulation. On the one hand, although the desire to be admitted to the target institution remains important as the examination approaches, the key to success in the final stage actually lies in completing the small tasks of each day. Yet some candidates become immersed in negative emotions generated by low late-stage efficiency and self-doubt. They are filled with anxiety and worry about the final result, remain preoccupied with unknown future events rather than focusing on daily micro-goals, and consequently sink into even greater anxiety. When negative emotions arise, they fail to intervene and adjust in a timely manner, allowing anxiety to expand continuously and affect final performance. One interviewee said that

“By the late stage, they had barely studied at all because they simply could not absorb anything; every day they only worried about whether they could get into graduate school, their concentration was inadequate, their efficiency was low, and they became even more anxious as a result”(F3E2ZB).

Affected by the employment choices of their peers, some candidates began to doubt the core value of taking the postgraduate entrance examination and the rationality of their own study goals. The essence of this cognitive fluctuation lies in the weakening of the candidates' belief in pursuing postgraduate studies and the instability of their intrinsic motivation.

“I took the postgraduate entrance examination actually to alleviate the employment pressure. When I saw others had found employment, I thought that I should have found employment first in the beginning” (M2A1ZB).

On the other hand, during the later preparation period, they often faced the dilemma of the mismatch between the efforts made and the actual feedback. After long-term study but with simulated scores not meeting expectations, These candidates found it difficult to perceive the actual progress toward their goals, which led them to fall into self-doubt. When the actual effect of the preparation did not reach the expected result and some classmates successfully found employment through public recruitment or recruitment for teaching positions, they began to doubt the significance of the postgraduate entrance examination and their future employment after getting a master's degree, forming a self-awareness that the goal was difficult to achieve. When the actual effects of preparation fail to meet expectations and some classmates successfully obtain employment through the civil-service examination or institutional recruitment, such candidates may begin to question the meaning of taking the postgraduate entrance examination and the employment value of a master's degree, thereby forming a self-cognition that the goal is difficult to achieve. These candidates tend to over-focus on mock scores while neglecting the active adjustment of test-taking mentality. They fail to recognize that mock scores are not the core variable determining the final examination outcome and do not treat mock examinations as a crucial auxiliary means of identifying knowledge gaps and optimizing test-taking strategies before the actual examination. As a result, the preparatory value of mock examinations is not effectively activated. One interviewee admitted that

“My mock examinations were very unsatisfactory. I felt that they knew the knowledge, but could not answer the questions, and therefore believed that they would be even less capable of answering the real examination questions and had no confidence in being admitted” (F2A2ZK).

**Figure 1:**
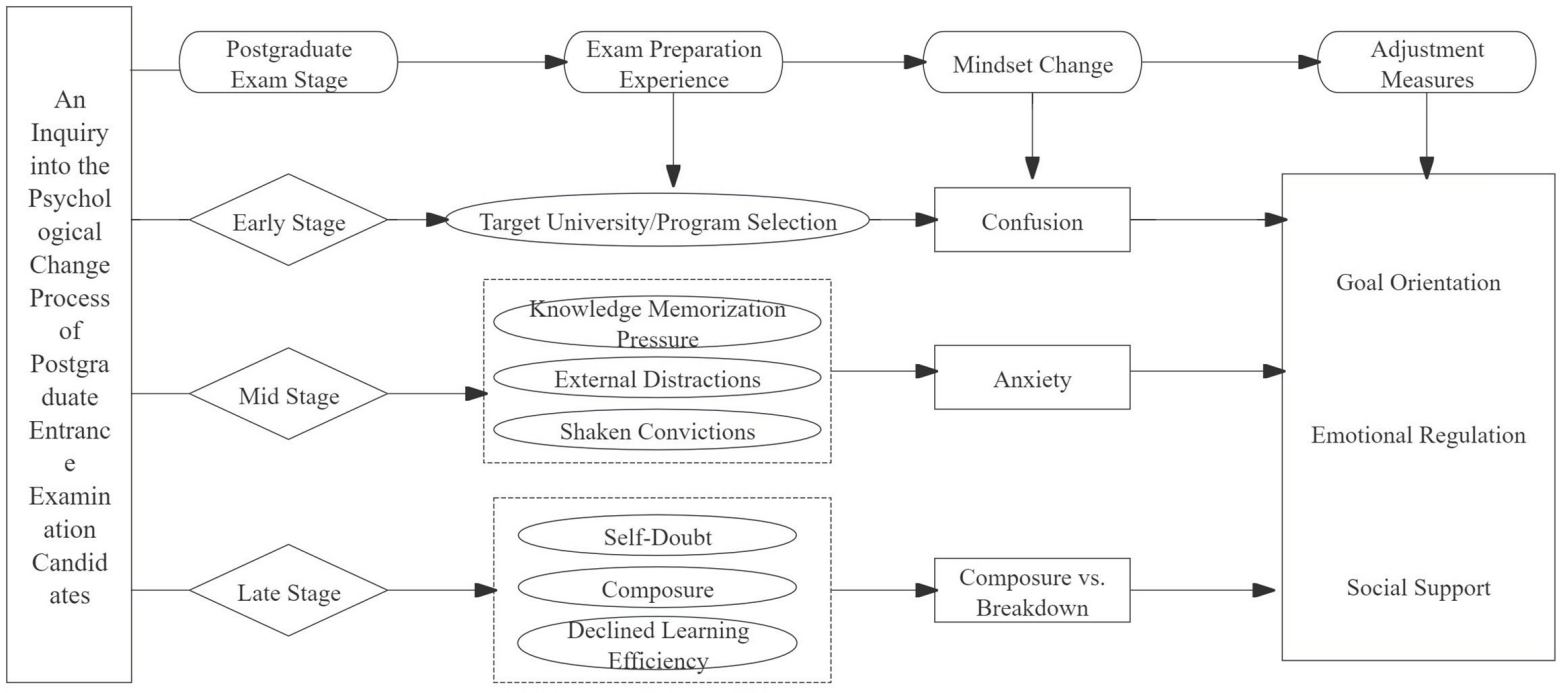
Analysis of the psychological growth process of postgraduate entrance examination candidates.

In conclusion, during the initial stage, postgraduate students become confused due to the complexity and variability of their target universities. In the middle stage, they experience anxiety caused by academic pressure, external interference, and wavering beliefs. In the later stage, they exhibit a bipolar state of composure and collapse. The deep psychological changes of these students stem from the interactive influence of goal orientation, emotion regulation, and social support. To systematically present this psychological growth trajectory and propose specific stage-based mental adjustment measures from the dimensions of goal anchoring, emotion regulation, and social support, it will help postgraduate students achieve psychological growth during the long preparation period and smoothly cope with challenges at each stage. For the specific framework of the psychological growth trajectory of postgraduate students and the stage-based mental adjustment measures, please refer to the writing structure shown in the Figure 1 to present the core logic in a more intuitive way.

## 5 Measures for mindset adjustment during preparation for the postgraduate entrance examination

### 5.1 Strategies for addressing early-stage confusion

Firstly, identify the core conditions and narrow down the scope of school selection. At the beginning of the postgraduate entrance examination, candidates often face the dual pressures of choosing a school and being overwhelmed by information, which leads to unclear goals and insufficient motivation. Candidates should consider various factors such as personal strength, career planning, and the strength of the school and its major, and determine the non-negotiable core conditions to quickly filter out a large number of options and make the goal clearer. First, select the major that interests you. Decide whether to continue studying in the same major or to take the exam for another major. If you are taking the exam for another major, you must assess your knowledge foundation, learning ability, and true interests. Avoid blindly following popular majors; if you are pursuing the same major, consult the faculty profiles, research projects, and laboratory facilities of each college to find the direction that best matches your academic interests. Second, determine the target city. The choice of location directly affects the convenience of future internships, employment, and life. Candidates must clearly define their expectations for the cities where they will study and develop in the future. Third, assess your own abilities. Refer to your GPA ranking during your undergraduate studies, your mastery of specialized courses, and your foundation in public courses such as English. This will give you an initial understanding of your position among all candidates in the country. Honestly assess your self-discipline, stress resistance, and ability to continuously learn, and use this to provide a reference for choosing a competitive top university or a stable university. Second, collect key information so as to match target institutions. After an initial scope has been outlined, Candidates need to conduct in-depth and precise intelligence analysis of candidate institutions in order to ensure information symmetry and avoid later failure caused by information errors. Specifically, candidates need to focus on key information and further narrow the range of choices. Once a general range of target institutions has been identified, candidates can obtain information by consulting official university websites, admissions brochures, postgraduate entrance examination forums, and senior students who are currently enrolled, and then select the institution that best fits their foundation and preparation capacity. For each candidate institution, they should focus on collecting and examining such key information from the past 3 years as re-examination score lines, applicant-to-admission ratios, and the number of recommended admissions; the reference books for specialized courses and the style of past examination questions; the elimination rate in the re-examination, and whether first-choice applicants are protected. This helps candidates judge competitiveness, assess preparation fit, clarify the characteristics of institutional admission rules, and thereby improve the probability of ultimate admission.

Finally, integrate information resources and lock in the final choice. At the decision-making stage, candidates need to weigh subjective preferences against objective data comprehensively and make a decision that they themselves find convincing and are unlikely to regret. Specifically, under the guidance of goal orientation, candidates should adopt rational analysis by creating a difficulty-analysis table for candidate institutions and, drawing on a three-tier strategy of reach, match, and safety choices, comprehensively assess both the fit of core conditions and the match of key information. They should then reassess the remaining secondary factors—such as geographic location, campus environment, and surrounding environment—in an integrated manner. Once the target institution has been determined, candidates should ensure the stability of their choice, concentrate on preparation, and avoid repeated adjustments and the internal depletion associated with decision-making.

### 5.2 Strategies for alleviating mid-stage anxiety

First, strengthen emotional-regulation training so as to prevent the generalization of negative emotions. The postgraduate entrance examination is a protracted struggle. Once the novelty of the early stage has faded, most candidates in the middle stage shift psychologically from early positivity and confidence to anxiety and confusion. Candidates should combine scientific learning methods with the construction of a stable psychological support system for preparation, so as to prevent negative emotions from diffusing unchecked. On the one hand, they should accept emotional fluctuations and reject perfectionism. When negative emotions such as anxiety and confusion arise, candidates should seek objective evidence in order to correct cognitive distortions and avoid emotional self-criticism or suppression. They should also provide reasonable channels for emotional release—for example, by seeking outside help or taking appropriate breaks—so that their mindset can be adjusted and brought back into alignment with the rhythm of study. On the other hand, they should focus on controllable factors and reduce the influence of uncontrollable disturbances. By using lists to distinguish the controllable from the uncontrollable, candidates can shift their attention away from uncontrollable matters such as changes in reference books and toward controllable process indicators, such as completing daily learning tasks with adequate quality and quantity. In this way, process management can replace result-oriented anxiety and preparation concentration can be improved.

Second, optimize time management in order to reduce the impact of external disturbances. Time management is essentially the scientific matching of limited resources with unlimited goals, and it requires individuals to possess a clear awareness of their own boundaries of ability and the allocation of their energy. By setting explicit schedules, individuals can break long-term goals down into operable short-term tasks and prevent external factors from eroding their goals. During preparation, candidates should optimize time management in accordance with their own circumstances. Specifically, they may record their study routines for 1 week, observe which periods of the day feature the clearest thinking and the highest degree of concentration, and allocate these “golden periods” to the subjects that require the deepest thinking. Likewise, they should identify lower-efficiency periods and assign to them mechanical or low-cognitive-load tasks such as copying notes, organizing incorrect-answer notebooks, or listening to politics audio materials. In addition, candidates should make more realistic estimates of study tasks. Rather than setting vague goals such as “study English all morning,” they should define concrete and operable tasks, such as memorizing a certain number of words, analyzing a reading-comprehension passage, or writing an essay, thereby laying the foundation for more precise planning in the next step.

Finally, actively seek social support to alleviate the pressure of exam preparation. When facing exam pressure, postgraduate students actively seek external support from their families, schools, etc. This is crucial for strengthening emotional regulation and maintaining the resilience of exam preparation. Family capital is an important factor for students to pursue their research interests (Cao and Zhang, 2026). Candidates need to actively build communication bridges and seek economic support from their families. When facing parents' confusion, they should have rational and honest communication. They can share their motivation for the exam and long-term career plans, and try to take the exam first and then re-enter the workforce if unsuccessful. This way, they can address parents' concerns about time costs and employment prospects, guide their emotional understanding and tolerance, and transform family pressure into a supportive force. At the school and internship unit levels, regarding the conflict between internship and the critical period of exam preparation, candidates can submit written applications to the school's academic affairs office and the college, explaining the urgency of the later exam review period, and applying for an elastic internship system to postpone or advance the internship time. For the determined internship unit and internship time, candidates can communicate with the responsible person in advance and apply to reduce the proportion of repetitive work and strive for fragmented study time. By actively communicating, they can reduce the interference of the internship on the exam preparation rhythm.

### 5.3 Strategies for coping with late-stage breakdown

Rekindle the original intention of your studies and enhance your motivation for the final push. During the final stage of the exam preparation, rekindling the original intention of your study is the key path to maintaining the internal motivation. When the review becomes weary, candidates can recall the original decision for taking the exam to stop the spread of negative cognition. Firstly, they can awaken the original intention of the goal by recalling the core demands for choosing to take the exam, clarify the deep connection between the goal and personal growth, and reconstruct the significance of the goal for themselves; Secondly, they can visualize the longing for the goal by imagining the learning environment of the ideal university, the research environment, or the development opportunities that can be gained with a postgraduate degree in the future, converting the abstract goal into a perceptible image, and stimulating the yearning for the goal and the desire for action; Thirdly, they can strengthen their determination toward the goal by reviewing the difficulties overcome for the goal during the review process, such as overcoming the problems with a large amount of knowledge memorization, breaking through weak subjects, balancing the relationship between review and internship, and using past persistence and progress to prove the value of persistence, reducing wavering during the final push, strengthening the motivation for continuous review, and providing stable psychological support for the final push stage.

Thoroughly carry out daily tasks and steadily advance the preparation. The execution and completion of tasks are the main factors contributing to the anxiety of candidates during the later stage of their preparation. The completion status of tasks can quantify students' mastery of knowledge and help them build confidence in advancing their preparation according to the plan and achieving results, forming a positive psychological cycle. At the task arrangement level, the principle of acting within one's capabilities should be followed. Firstly, through goal decomposition, the macroscopic preparation tasks are transformed into single, actionable specific actions to avoid the pressure of overly broad tasks; secondly, adjust the tasks dynamically based on the completion status of the previous day's tasks. If most tasks are not completed, reduce the number of tasks for the current day; if all tasks are completed ahead of schedule and the energy is sufficient, slightly increase the number of tasks to avoid a mismatch between task volume and actual ability; finally, reserve some study time as an elastic space for handling unfinished tasks or dealing with unexpected situations, to prevent anxiety caused by overly full plans.

## 6 Research findings and discussion

### 6.1 Research findings

This article, by analyzing the preparation experiences of postgraduate entrance examination candidates, has achieved the preset research objectives. Based on the research process and results, the following conclusions are drawn: During the preparation period, all postgraduate entrance examination candidates are under varying degrees of pressure. The difficulties faced by students at different stages are also different, and this difference leads to the dynamic changes in students' psychological states throughout the preparation period. These changes occur throughout the entire preparation process and are constantly adjusted according to the progress of the preparation, the degree of knowledge mastery, and the influence of external environment. The study also finds that candidates taking the examination for the first time and those taking it for a second or subsequent time experience different forms of psychological pressure. Specifically, first-time candidates' pressure is concentrated mainly at the knowledge level, arising from unfamiliarity with the body of knowledge required for the examination, insufficiently developed preparation systems, and a lack of familiarity with the examination process and patterns of question setting, all of which lead to heavy pressure in knowledge accumulation and test preparation. By contrast, the pressure experienced by second-time candidates is primarily mental and emotional, and is manifested chiefly in self-doubt brought about by previous failure in preparation; the instability of examination questions and the cost of time make their margin for error relatively smaller. Consequently, their psychological burden is even heavier. Overall, students preparing for the postgraduate entrance examination more or less experience a certain degree of psychological pressure, and the pressure they bear with respect to school selection, the written examination, and the interview is closely related to China's postgraduate admissions and selection system. The pressure associated with preparation stems largely from shortcomings in the current examination system in three respects. First, the nationwide unified examination limits the mechanism of school selection: candidates are given only one initial chance to choose a target institution, and although adjustment channels exist later, the opportunities are limited and the risks remain high, which directly increases both choice pressure and psychological burden. Second, the rules of assessment and selection in the admissions system are not fully rational. Although many institutions nominally allocate 50% of the overall score to the written examination and 50% to the interview, interviews are conducted on a merit basis, which means that even candidates who reach the national score line may still lose the opportunity to enter the interview because their scores are only marginally above the line. This forces all candidates to chase very high scores merely in order to secure an interview ticket, thereby intensifying preparation pressure invisibly. Third, the timing of admission is not optimally arranged. Under the current process, registration begins in the second half of September each year, the written examination is held at the end of December, results are released in February of the following year, re-examinations take place in April, adjustment begins in the second half of April, and admissions are finalized only at the end of May. Written examination results are released relatively late, and both interviews and adjustment take a long time. Given that most institutions invite roughly three interviewees for every place, two-thirds of those who secure an interview opportunity will ultimately still be eliminated. Candidates must therefore invest substantial time and energy in interview preparation, yet by the time they are confirmed as not admitted, little time remains to prepare for spring recruitment, and they may even miss the best employment opportunities altogether. In other words, candidates may simultaneously lose both the opportunity to upgrade their educational credentials and the opportunity to obtain employment, which further magnifies their psychological pressure and preparation burden.

### 6.2 Discussion

Japan and China have similar systems for graduate entrance examinations, both setting up two stages for graduate admission: written tests and interviews. China adopts a nationwide unified proposition mechanism and a centralized quota allocation system for postgraduate recruitment, which restricts universities' autonomy to a large extent. Coupled with the rigid examination schedule and limited options for candidates in selecting target universities, this institutional design has to some extent aggravated postgraduate applicants' mental stress during their preparation period. In contrast, Japan practices a university-autonomous enrollment system, where the authority of exam proposition is delegated to individual institutions. Featuring a flexible examination timetable and diverse choices for candidates, this system also attaches greater weight to interview performance and process-oriented assessment in its evaluation framework. Such an assessment model not only eases the mental pressure on candidates, but also helps universities identify and recruit talents with genuine research potential. Therefore, drawing on the rational elements of Japan's postgraduate admission system can provide valuable insights for the optimization of China's postgraduate recruitment mechanism, thereby promoting the high-quality development of postgraduate education in China.

1. Japan's Postgraduate Admissions Examination System

Japan is selected as a comparative case for examining students' psychological pressure under postgraduate admissions systems for a core reason: both Japan and China attach great importance to the talent-selection function of higher education, and postgraduate admissions in both countries include two core components—written examinations and interviews—which provides a feasible basis for comparison. A comparison readily shows that, although the core components of postgraduate admissions are similar in the two countries, there are significant differences in implementation procedures and selection standards. It is precisely this differentiated institutional design that makes Japanese students' psychological pressure lower than that of Chinese students during postgraduate application and preparation. Exploring the internal relationship between Japan's admissions design and students' psychological pressure can therefore provide important theoretical and practical reference points for alleviating the psychological pressure of China's postgraduate applicant population and optimizing the existing admissions system.

The graduate admission examination system in Japan mainly consists of two parts: written tests and interviews. However, the pressure on Japanese students to become graduate students is relatively low. This is specifically manifested as follows: Firstly, the highly autonomous admission model of universities effectively disperses the risks of pursuing higher education. Japanese universities have considerable autonomy in graduate admission. They conduct their own examination and admission, without a national unified examination. Each university can independently set admission standards. In this regard, students can apply to multiple universities simultaneously. Even if one application fails, it will not affect their ability to apply to other universities. The tolerance rate for admission is high, avoiding the anxiety of being determined by one exam and ending with failure. There is no psychological burden of having to start over if one fails. Moreover, the number of admission places is determined by professors based on research needs, without strict fixed restrictions. This further reduces the intensity of competition and the pressure of internal competition. Second, selection criteria are not purely examination-oriented, which weakens the decisive role of scores. In Japan's postgraduate admissions process, selection is not guided solely by test scores; instead, research potential is treated as the core criterion. Candidates' practical ability in conducting paper-based research and scientific experiments is a key basis for judging scientific potential [20]. On this foundation, professors further assess candidates' logical thinking and language expression through the quality of their research proposals and their interview performance. In terms of score weighting, interviews generally account for a greater share of the total score, while written examination results need only reach a passing standard, which to some extent reduces candidates' pressure to engage in high-intensity test-oriented preparation. Third, the system of professors' informal prior acceptance and the availability of flexible progression pathways reduce psychological anxiety. In the Japanese context, informal prior acceptance refers to a professor's private commitment to accept a student's postgraduate application. Because such a system is widely practiced in Japan, candidates can communicate with professors in advance, enabling both sides to gain mutual understanding in terms of personality and research focus. Once informal acceptance is obtained, the subsequent written examination and interview often serve more as procedural confirmation, the probability of admission becomes high, and uncertainty-related confusion is reduced. In addition, the preparatory student system provides a buffer for candidates: after a preparatory period, the probability of passing the master's examination is much higher than for direct applicants, and even if candidates fail, they still have alternative pathways and therefore do not have to bear excessive pressure.

Overall, Japanese students preparing for postgraduate study tend to display composure and low anxiety. This is not because they possess stronger stress tolerance, but because the admissions system achieves institutional decompression by dispersing risk, weakening test orientation, and providing flexible pathways. Such a design not only helps select talents with research potential but also takes students' psychological needs into account, thereby offering important reference for the optimization of China's postgraduate admissions system.

2. Implications of Japan's Postgraduate Admissions System for China

(1) Grant Universities Greater Admissions Autonomy

Taking into account the advantages of independent admissions in Japanese universities, it is appropriate to appropriately delegate the autonomy of postgraduate admissions to universities, granting them more decision-making space in the admissions process. Break away from the current single model of unified examination questions and unified score lines, and allow universities to set their own examination questions based on their own disciplinary characteristics and training goals, and flexibly determine the admission standards. At the same time, relax the restrictions on candidates' applications, allowing them to submit applications to multiple universities simultaneously, to disperse the risks of further education and reduce the psychological pressure caused by the “one exam determines life” and “failure in one university means losing all opportunities” scenarios. Universities can independently adjust the weights of written tests and interviews according to disciplinary needs, avoiding a “one-size-fits-all” approach in the admissions process. This can not only enhance the flexibility of admissions but also enable universities to more accurately select students who meet their own training requirements, achieving a precise connection between admissions and training.

(2) Optimize Examination Scheduling

Based on the rationality of the graduate student admission schedule in Japan, optimize the time nodes for the national postgraduate entrance examination and admission in China, alleviate the dual pressure on candidates of preparing for the exam and seeking employment. Appropriately advance the dates of the preliminary and final examinations, shorten the period from the release of scores to the final admission, and avoid the current situation where admissions are completed only in April or May each year. Regarding the problem of a high interview ratio and the loss of employment opportunities for failed candidates, reasonably compress the preparation and implementation time for interviews, clearly define the time limit for announcing the interview results, and reserve a sufficient spring recruitment buffer period for failed candidates. At the same time, coordinate the time for the postgraduate entrance examination and the spring recruitment, reduce the conflicts between the two, and allow candidates not to be overly troubled between preparing for the interview and seeking employment, effectively reducing the psychological burden caused by employment uncertainty, and achieving a positive connection between further education and employment.

(3) Adopt Diversified Admission Criteria

Drawing on Japan's admissions philosophy of taking research potential as the core criterion, China could improve diversified admission standards and standardize the allocation of weight between written examinations and interviews. Although most Chinese institutions stipulate that written examination results and interview results each account for 50% of the total score, in actual implementation the weight of the written examination often remains implicitly too high, and the tendency toward “scores-only evaluation” has not been fully overcome. Accordingly, the 50:50 weighting between written examination and interview should be strictly implemented, and a “blind interview” system could also be introduced, under which interviewers are not informed of candidates' written examination scores during the interview stage, thereby preventing written examination scores from influencing interviewers' subjective judgment and ensuring the objectivity and fairness of interview evaluation. In addition, current contact and mutual understanding between teachers and students remain rather limited. It is difficult to comprehensively grasp both sides' needs and fit through only one or two encounters during the interview itself, which is not conducive to two-way matching between supervisors and students. More communication opportunities should therefore be incorporated into the admissions process, allowing candidates to learn in advance about supervisors' research directions and training philosophies, and enabling supervisors to gain a fuller understanding of candidates' research interests and personal characteristics. Such changes would facilitate two-way matching between students and supervisors during admissions and thereby improve both admissions quality and cultivation outcomes.

## 7 Research contributions

Based on the theory of psychological resilience, this paper focuses on the psychological development process of postgraduate entrance exam candidates during their preparation period. Interviews with a number of candidates reveal that the preparation process can be divided into three phases: the preliminary stage, the intermediate stage, and the final stage. Meanwhile, the three dimensions of psychological resilience—goal orientation, emotional regulation, and social support—are embedded into different phases of the preparation process, on the basis of which a three-phase framework for candidates' preparation is constructed, namely initial confusion, intermediate anxiety, and final composure intertwined with breakdown.

## Data Availability

The original contributions presented in the study are included in the article/supplementary material, further inquiries can be directed to the corresponding author.
